# LPAAT3 incorporates docosahexaenoic acid into skeletal muscle cell membranes and is upregulated by PPARδ activation[S]

**DOI:** 10.1194/jlr.M077321

**Published:** 2017-12-28

**Authors:** William J. Valentine, Suzumi M. Tokuoka, Daisuke Hishikawa, Yoshihiro Kita, Hideo Shindou, Takao Shimizu

**Affiliations:** Department of Lipid Signaling,* National Center for Global Health and Medicine, Shinjuku-ku, Tokyo 162-8655, Japan; Departments of Lipidomics† University of Tokyo, Bunkyo-ku, Tokyo 113-0033, Japan; Lipid Science,** University of Tokyo, Bunkyo-ku, Tokyo 113-0033, Japan; Life Sciences Core Facility,§ Graduate School of Medicine, University of Tokyo, Bunkyo-ku, Tokyo 113-0033, Japan; Japan Agency for Medical Research and Development (AMED)†† Chiyoda-ku, Tokyo 100-0004, Japan

**Keywords:** omega-3 fatty acids, lipidomics, fatty acid/metabolism, lysophosphatidic acid acyltransferase 3, exercise, mass spectrometry, 1-acylglycerol-3-phosphate *O*-acyltransferase

## Abstract

Adaption of skeletal muscle to endurance exercise includes PPARδ- and AMP-activated protein kinase (AMPK)/PPARγ coactivator 1α-mediated transcriptional responses that result in increased oxidative capacity and conversion of glycolytic to more oxidative fiber types. These changes are associated with whole-body metabolic alterations including improved glucose handling and resistance to obesity. Increased DHA (22:6n-3) content in phosphatidylcholine (PC) and phosphatidylethanolamine (PE) is also reported in endurance exercise-trained glycolytic muscle; however, the DHA-metabolizing enzymes involved and the biological significance of the enhanced DHA content are unknown. In the present study, we identified lysophosphatidic acid acyltransferase (LPAAT)3 as an enzyme that was upregulated in myoblasts during in vitro differentiation and selectively incorporated DHA into PC and PE. LPAAT3 expression was increased by pharmacological activators of PPARδ or AMPK, and combination treatment led to further increased LPAAT3 expression and enhanced incorporation of DHA into PC and PE. Our results indicate that LPAAT3 was upregulated by exercise-induced signaling pathways and suggest that LPAAT3 may also contribute to the enhanced phospholipid-DHA content of endurance-trained muscles. Identification of DHA-metabolizing enzymes in the skeletal muscle will help to elucidate broad metabolic effects of DHA.

Skeletal muscle is highly adaptive tissue and can adjust its metabolic capacity in response to physiological demands. The adaptive response of skeletal muscle to endurance exercise involves a switch of glycolytic to oxidative fibers (fast to slow transition) that is associated with favorable whole-body metabolism including improved glucose control and resistance to obesity (1). Adaption of muscle to endurance exercise is transcriptionally mediated. The nuclear receptor PPARδ was the first identified transcriptional regulator of the adaptive response. With training, mice expressing constitutively active PPARδ in skeletal muscle could run twice as far as wild-type littermates (2). AMP-activated protein kinase (AMPK) is activated in response to exercise and is a key regulator of cellular metabolism. AMPK controls activities of several transcriptional regulators including PPARγ coactivator 1α (PGC1α), which coactivates PPARδ to cooperatively induce expression of genes that promote oxidative metabolism and fast to slow transition (1).

Omega-3 (n-3) fatty acids are widely taken as dietary health supplements, and DHA (22:6n-3) may have broad effects to promote metabolic health (3). Several reports indicated that enhanced incorporation of DHA into skeletal muscle phospholipids may occur during the adaptive response to endurance exercise (4–7). Enhanced phospholipid-DHA content in skeletal muscle was associated with increased oxidative fiber content and fast to slow transitioning of glycolytic fibers to more oxidative fiber types (4, 7, 8), suggesting that DHA content may have a functional role in promoting an endurance phenotype. In support of this theory, dietary DHA supplementation increased phospholipid-DHA content and enhanced gains of endurance in exercised rats (9). Also, in birds, either DHA or EPA (22:5n-3) supplementation strongly stimulated oxidative capacity of flight muscles of confined quail, concurrent with enhanced DHA or EPA levels in phospholipids (10).

Omega-3 fatty acids like α-linolenic acid (18:3n-3) cannot be synthesized in animals and are obtained by diet. Conversion of α-linolenic acid to DHA occurs at low levels, and dietary DHA intake is a primary determinant of phospholipid-DHA content. However, although the mechanisms are unknown, the enhanced phospholipid-DHA content reported in endurance-trained skeletal muscle occurred independently of dietary DHA intake (4–7). Senoo et al. (4) showed that mice which overexpressed PGC1α in skeletal muscle had enhanced levels of several DHA-containing phospholipid species, which were also upregulated by endurance training in a PGC1α-dependent process. Their study demonstrated that exercise-activated transcriptional programs may mediate enhanced DHA content in cellular phospholipids; however, the DHA-metabolizing enzymes involved in this process have not been identified. The biological significance of the enhanced DHA content also requires elucidation. DHA incorporation into phospholipids may alter biophysical properties of membranes as well as alter levels of free fatty acid DHA, and either of these may impact cellular metabolism.

In order to elucidate the biological significance of the enhanced DHA content in endurance-trained muscle, it is necessary identify the molecular entities that mediate DHA incorporation into skeletal muscle phospholipids and how their activities are regulated. In cellular membranes, lysophosphatidic acid acyltransferases (LPAATs) generate phosphatidic acid (PA), including DHA-containing PA, which is converted to other phospholipids including phosphatidylcholine (PC) and phosphatidylethanolamine (PE) (11). The purpose of the present study was to identify enzymes that catalyze DHA incorporation into skeletal muscle phospholipids and to understand how their activities may be regulated by exercise-induced signaling pathways. Our results indicate that LPAAT3 was upregulated and functioned to incorporate DHA into skeletal muscle cells during in vitro differentiation. This activity was enhanced by activation of the PPARδ and AMPK pathways and might be relevant to the increased DHA content of endurance-trained muscle.

## MATERIALS AND METHODS

### Reagents

DHA, arachidonic acid (AA; 20:4n-6), and linoleic acid (LA; 18:2n-6) were obtained from Cayman Chemicals and prepared as 100 mM stocks in ethanol. ON-TARGETplus siRNAs were purchased from Dharmacon. RNAiMAX was purchased from Invitrogen. The 5-aminoimidazole-4-carboxamide ribonucleotide (AICAR), collagenase (C6885), and type B bovine gelatin were purchased from Sigma-Aldrich (St. Louis, MO). GW501516 (GW1516) was purchased from R&D Systems (Minneapolis, MN). Dispase I was purchased from Wako Pure Chemicals and complete protease inhibitor cocktail was from Roche Applied Science.

### C2C12 cell culture

C2C12 cells were obtained from RIKEN and cultured at 37°C in a humidified atmosphere of 5% CO_2_. C2C12 cells were passaged in growth medium consisting of DMEM (Gibco) supplemented with 10% heat-inactivated (56°C, 30 min) FBS (Gibco) and 2 mM glutamine. To induce differentiation, the cell medium was replaced with differentiation medium consisting of DMEM containing 2% heat-inactivated horse serum (Gibco) and 2 mM glutamine. The differentiation medium was replenished daily for 7 days, and then the differentiated C2C12 myotubes were selectively enriched from undifferentiated cells based on adherence properties (12, 13). In brief, myotubes were detached in diluted trypsin/EDTA (0.05/0.002%) and preplated on 10 cm culture dishes for 30–60 min in growth medium to remove rapidly adhering undifferentiated cells. The unattached myotubes were collected, pelleted by centrifugation (240 *g*, 5 min), resuspended in differentiation medium, and plated onto 24-well Corning tissue culture plates, which had been coated with 0.1% gelatin (1 h at 37°C) and rinsed in DMEM. Undifferentiated C2C12 cells were also plated in growth medium (20,000 cells per well), and cell extracts were collected 2 days later for analysis by RT-PCR or Western blot.

### Mice

C57BL/6N mice were obtained from Clea Japan, Inc. (Tokyo, Japan). All research was conducted in accord with the Public Health Service policy on the Humane Care and Use of Laboratory Animals. Maintenance of the animal facility and use of animals was in full compliance with the Ethics Committee for animal experiments of the National Center for Global Health and Medicine, Tokyo, Japan.

### Isolation and culture of satellite cells

Satellite cells were isolated from the hind limbs of 4- to 12-week-old male C57BL/6N mice according to the method of Rando and Blau (14), with modifications. Hind limb muscles were collected, rinsed in PBS, and minced in PBS supplemented with 5 mM CaCl_2_, 5 mM collagenase, and Dispase I (400 protease units per milliliter). The minced tissue was incubated at 37°C and triturated every 30 min. After 90 min, the digested slurry was diluted with PBS and passed through 100 μm and then 40 μm cell strainers (Falcon). The filtrate, containing cells and tissue debris, was centrifuged at 350 *g* for 15 min and the pellet was resuspended in growth medium consisting of Ham’s F-10 medium (Gibco) supplemented with 20% heat-inactivated FBS, 1% antibiotic antimycotic (Sigma), and 5 ng/ml basic fibroblast growth factor (R&D Systems). The cells were cultured on petri dishes (Rikaken; RSU-SD9015-2), which had been coated with type I collagen (Sigma; C8919). Culture media were replaced every 2–3 days, and cells were maintained at less than 70% confluency. Myoblasts were enriched during passaging by selective detachment in PBS and preplating to remove contaminating fibroblasts (15). Satellite cells were used for experiments within 10 passages.

### Satellite cell differentiation

Satellite cells were seeded onto type I collagen-coated 24-well plates (Iwaki; 4820-010) in growth medium and allowed to attach overnight. To induce differentiation, growth medium was replaced with low serum differentiation medium consisting of DMEM containing 2% heat-inactivated horse serum, 1% chick embryonic extract ultrafiltrate (US Biological Life Sciences), and 2 mM glutamine. The medium was replenished daily during differentiation. Where indicated in the text, media were supplemented with a fatty acid mixture comprised of 5 μM each of LA, AA, and DHA to supply substrates for changes in cellular phospholipid compositions.

### siRNA transfection of satellite cells

ON-TARGET plus siRNAs targeting murine LPAAT3, PPARδ, and control siRNA were purchased from Dharmacon. The individual siRNAs used were J-059804-09 (siLPAAT3 #1), J-059804-12 (siLPAAT3 #2), J-042751-09 (siPPARδ #1), and J-042751-10 (siPPARδ #2). Nontargeting Pool siRNA D-001810-10-20 was used as a control (siCONTROL). Satellite cells were transfected using RNAiMAX transfection reagent (Invitrogen) in 24-well collagen-coated tissue culture plates (Iwaki), with 1.5 μl RNAiMAX and 5 pmol siRNA applied per well. For LPAAT3 knockdown, siRNAs and RNAiMAX transfection reagent were complexed in DMEM and applied to cells at the onset of differentiation protocols. In the case of PPARδ knockdown, siRNAs were complexed with RNAiMAX in Ham’s F-10 medium and applied to cells in growth medium lacking antibiotics; after 16 h, culture medium was replaced with differentiation medium and cells were allowed to differentiate for 48 h before RNA extracts were collected for analysis.

### Drug treatment of satellite cells

GW1516 was prepared as a stock solution in DMSO and AICAR was dissolved in DMEM. Differentiation media, including any fatty acid supplementation and drug compounds, were replenished daily during differentiation protocols. For GW1516 dose response and time course, GW1516 was applied to satellite cells at the onset of differentiation and replenished daily in fresh medium. To detect GW1516- and AICAR-mediated changes in cellular phospholipids, satellite cells were cultured for 2 days in differentiation medium without fatty acid supplementation or drug compounds. Two days after the onset of differentiation, cellular media were replaced with fresh differentiation medium that was supplemented with the fatty acid mixture and also contained vehicle, GW1516 (1 μM), and/or AICAR (1 mM). Fresh differentiation media with fatty acid mixture and compounds were again replenished the next day, and extracts were collected the following day (day 4 of differentiation) for LC-MS and RT-PCR analyses.

### Light microscopy

Images of live C2C12 and satellite cells were captured on an Olympus CKX41 inverted microscope equipped with a DP20 microscope camera using Olympus CAch N 10× objective lens.

### RT-PCR

Total cellular RNA from C2C12 or satellite cells was isolated using an RNeasy mini kit (Qiagen, Valencia, CA). cDNAs were synthesized using SuperScript III reverse transcriptase and random primers (Invitrogen). RT-PCR analysis was performed using a StepOnePlus real-time PCR system and Fast SYBR Green Master Mix (Applied Biosystems). mRNA expression was normalized to 18S rRNA and fold-changes were calculated using the 2^−ΔΔCT^ method. The PCR primer sequences are shown in **Table 1**.

**TABLE 1.** List of primers used for RT-PCR

Gene	Forward Primer	Reverse Primer
LPAAT1	AAACGAGGCGCCTTCCA	GGAGTAGAAGTCTTGATAGGAGGACATG
LPAAT2	TGTGGGCCTCATCATGTACCT	AGGTCGGCCATCACAGACA
LPAAT3	AAGCACCTATACCGCCGTATCA	GACCACCACTCCAGGAGCAT
LPAAT4	AAGCAGCTGTTCCGCAAGA	CCACCACTCCAGAAGCATCA
AGPAT5	AATGAGAAAGGTTCAGGAAAATACTCA	TGAATATGAAGTTTTGGGCACTGT
Pax7	TCAAGCCAGGAGACAGCTTG	TGTGGACAGGCTCACGTTTT
MyoD	AGCATAGTGGAGCGCATCTC	GTTCCCTGTTCTGTGTCGCT
Myogenin	GTGAATGCAACTCCCACAGC	CCACGATGGACGTAAGGGAG
PPARδ	TCTCCCAGAATTCCTCCCCT	GAGCTTCATGCGGATTGTCC
PDK4	AAAGATGCTCTGCGACCAGT	GGGTCAAGGAAGGACGGTTT
UCP3	GAAAGGGACTTGGCCCAACA	TCTTTACCACATCCACCGGG
18S	CTCAACACGGGAAACCTCAC	AGACAAATCGCTCCACCAAC

### Western blots

C2C12 or satellite cells were disrupted on ice in 1 ml of homogenization buffer [100 mM Tris-HCl (pH 7.4), 300 mM sucrose, and 1× complete protease inhibitor cocktail] using a tight Dounce homogenizer followed by a probe sonicator (Ohtake Works), and then centrifuged at 800 *g* for 10 min to pellet unbroken cells and debris. The supernatants were centrifuged at 100,000 *g* for 1 h at 4°C, and resultant pellets were resuspended in 2× SDS-gel-loading buffer [4% SDS, 20% glycerol, 0.125 M Tris (pH to 6.8)]. Protein concentrations were determined using a Pierce BCA protein assay kit (Thermo Fisher Scientific). The 2-mercaptoethanol (5% final concentration) and bromophenol blue were added to the samples. After incubation at room temperature for at least 30 min, equal amounts (4–10 μg) were resolved by 8% SDS-PAGE and then transferred to a Hybond ECL nitrocellulose membrane (GE Healthcare) using a Trans-Blot SD transfer cell (Bio-Rad). Membranes were blocked in 5% skim milk in TBS containing 0.1% Tween 20 (TBS-T) overnight at 4°C. Membranes were probed with an anti-mouse LPAAT3 antibody (16) at room temperature for 60 min, washed three times for 5 min each with TBS-T, and then incubated with horseradish peroxidase-conjugated anti-rabbit IgG (GE Healthcare) for 30–60 min. Detection was performed with ECL Select reagent and imaged using an ImageQuant LAS 500 (GE Healthcare). To confirm equal loading of the samples, membranes were reblocked overnight and then probed with anti-calnexin (#610523; BD Transduction Laboratories) followed by horseradish peroxidase-conjugated anti-mouse IgG (GE Healthcare).

### Phospholipid analysis by LC-MS

Satellite cells were cultured on 24-well plates and cellular phospholipids were extracted using methanol (500 μl/well) (17). After incubation for 5 min at room temperature, the extracts were centrifuged at 4°C for 10 min at 18,700 *g*, and supernatants were collected and further diluted in methanol before LC-MS analysis. LC-selected reaction monitoring (SRM)-MS analyses were performed using a Nexera UHPLC system and LCMS-8040 or -8060 triple quadrupole mass spectrometers (Shimadzu). An Acquity UPLC BEH C8 column (1.7 μm, 2.1 × 100 mm; Waters) was used with the following ternary mobile phase compositions: 5 mM NH_4_HCO_3_/water (mobile phase A), acetonitrile (mobile phase B), and isopropanol (mobile phase C). The pump gradient [time (%A/%B/%C)] was programmed as follows: 0 min (75/20/5) to 20 min (20/75/5) to 40 min (20/5/75) to 45 min (5/5/90) to 50 min (5/5/90) to 55 min (75/20/5). The flow rate was 0.35 ml/min and column temperature was 47°C. Injection volume was 5 μl. Comprehensive LC-SRM-MS analysis was performed in the positive ion mode electrospray ionization with the transitions [M+H]^+^ → 184 for PC and [M+H]^+^ → [M+H-141]^+^ for PE to detect all diradyl PC and PE species possessing even number carbon chains 14–24 carbons in length in sn-1 and sn-2. In some cases, negative ion mode was used to resolve individual acyl chain identities (see below). Signals obtained in positive ion mode yielded superior dynamic range and were used for all comparative analyses. Chromatographic peak areas were used for comparative quantitation of each molecular species (e.g., 38:6) within a given class (e.g., PC). Peak areas of each individual species were normalized against the sum of all peak areas within that class to determine the relative abundances (expressed as percent of total), and all major species (comprising at least 2% of total PC or PE signals) were plotted.

To characterize the acyl-chain compositions of DHA-containing species, targeted LC-SRM-MS/MS using both positive and negative ion modes was performed on PC(38:6), PC(40:6), PC(40:7), PE(38:6), PE(40:6), and PE(40:7). The chromatographic peaks in positive ion mode with differing retention times were obtained with the following transitions: [M+H]^+^ → 184 for PC and [M+H] ^+^ → [M+H-141]^+^ for PE. Negative ion mode fragment ion signals of possible fatty acyl chains were obtained using the following transitions: [M + HCO_3_]^−^ → [FA-H]^−^ for PC and [M-H]^−^ → [FA-H]^−^ for PE, where [FA] is the monoisotopic mass of the fatty acid. The following acyl chains were targeted: C16:0 (*m/z* 255.1), C16:1 (*m/z* 253.1), C18:0 (*m/z* 283.1), C18:1 (*m/z* 281.1), C18:2 (*m/z* 279.1), C18:3 (*m/z* 277.1), C20:1 (*m/z* 309.1), C20:2 (*m/z* 307.1), C20:3 (*m/z* 305.1), C20:4 (*m/z* 303.1), C20:5 (*m/z* 301.1), C22:3 (*m/z* 333.1), C22:4 (*m/z* 331.1), C22:5 (*m/z* 329.1), C22:6 (*m/z* 327.1), C24:5 (*m/z* 357.1), and C24:6 (*m/z* 355.1). Of note, *m/z* 283.1 may contain signals of decarboxylated C22:6 (DHA) in addition to C18:0 signals; however, this did not hinder fatty chain identifications of the targeted species.

### Statistical analysis

All statistical analyses were performed using the means of at least three independent experiments, each performed in triplicate, using GraphPad PRISM5 or PRISM6 software (GraphPad Software, La Jolla, CA). RT-PCR expression values were log transformed before statistical analyses (18). Student’s *t*-tests (paired, two-tailed), one-way ANOVA (Tukey’s posttests, repeated measures), and two-way ANOVA (Bonferonni posttests, repeated measures) were performed and are indicated in the figure legends. *P* values <0.05 were considered statistically significant.

## RESULTS

### Changes in LPAAT expression during differentiation of C2C12 cells

**Fig. 1.**
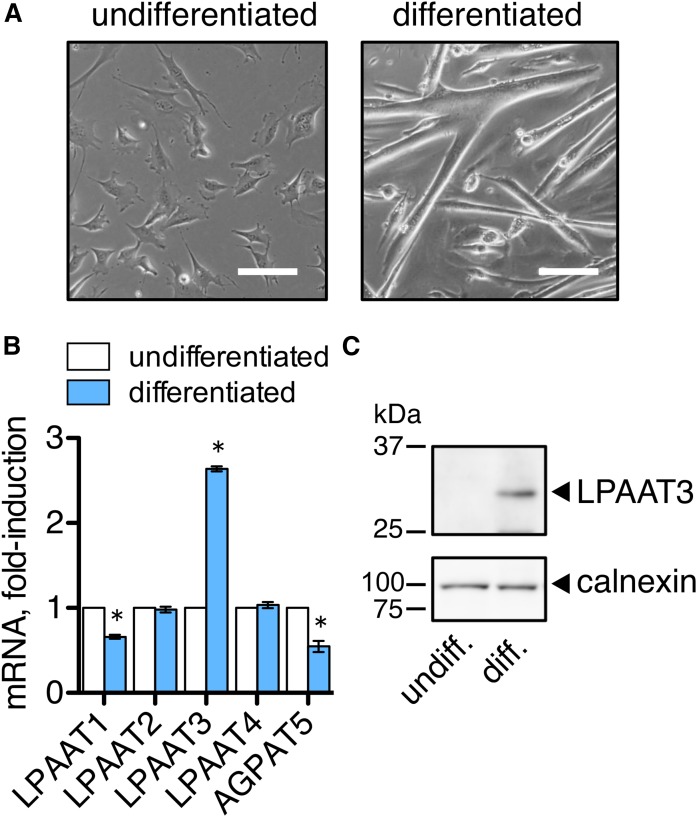
LPAAT3 was upregulated during differentiation of the C2C12 myoblast cell line. C2C12 cells were differentiated in low serum medium for 7 days, and then the myotubes were collected and differentiated for an additional 2 days. Undifferentiated cells were cultured in growth medium. A: Images of undifferentiated and differentiated C2C12 cells are shown. Scale bar = 100 μm. B: LPAAT3 mRNA was increased during differentiation. Expression levels were measured by RT-PCR. Data are expressed as the mean ± SEM from three independent experiments. *P < 0.05; *t*-test. C: LPAAT3 protein levels increased during differentiation. LPAAT3 was detected by Western blot analyses. Calnexin was detected as a loading control. Representative blots of three independent experiments are shown.

Four LPAAT enzymes (LPAAT1–4) and another candidate enzyme, 1-acylglycerol-3-phosphate *O*-acyltransferase (AGPAT)5, have been identified that function in the de novo phospholipid synthesis pathway (19–23). These LPAAT enzymes vary in their expression patterns and substrate specificities and provide diversity to membrane phospholipids. In order to examine whether LPAATs might function to remodel cellular membranes during skeletal muscle differentiation, we measured LPAAT1–4 and AGPAT5 mRNA levels in the murine C2C12 skeletal muscle cell line. C2C12 cells differentiate and fuse to form multinuclear myotubes when cultured in low serum media. We compared LPAAT expression levels in undifferentiated C2C12 cells to levels in the differentiated myotubes (**Fig. 1A**). LPAAT3 mRNA was 2- to 3-fold upregulated in the differentiated C2C12 cultures, while other LPAATs were unchanged or decreased (Fig. 1B). LPAAT3 protein levels were also markedly increased following differentiation (Fig. 1C).

### Changes in LPAAT expression during differentiation of satellite cells

**Fig. 2.**
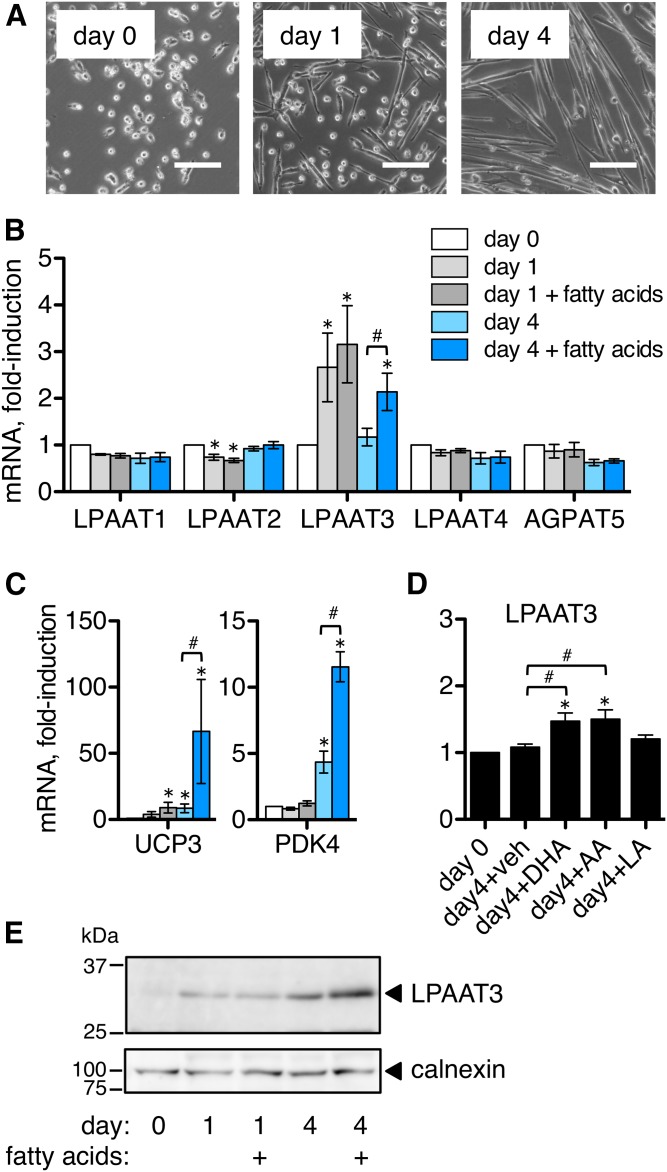
LPAAT3 levels were upregulated during satellite cell differentiation, and expression was enhanced by fatty acid supplementation. Satellite cells were differentiated in low serum medium, and some media were supplemented with a fatty acid mixture of LA, AA, and DHA (5 μM each). mRNA expression levels were measured by RT-PCR and protein levels were measured by Western blot. A: Images of satellite cells during differentiation. Satellite cells elongated and began fusing by day 1 and formed multinucleated myotubes by day 4. Scale bar = 100 μm. B–D: mRNA expression levels during satellite cell differentiation. B: LPAAT3 mRNA peaked at day 1 and expression was enhanced at day 4 by fatty acid supplementation. C: PPAR target genes UCP3 and PDK4 were also upregulated by fatty acid supplementation, suggesting possible PPAR activation. D: Components of the fatty acid mixture were applied individually (5 μM) during 4 days of differentiation. Either DHA or AA enhanced LPAAT3 expression. E: LPAAT3 protein increased during satellite cell differentiation and levels were enhanced by fatty acid supplementation at day 4. Representative Western blot from three independent experiments is shown; calnexin was detected as a loading control. RT-PCR data represent the mean ± SEM from three or four independent experiments. *P < 0.05, differentiated versus undifferentiated; ^#^*P* < 0.05, fatty acid supplementation versus no supplementation; one–way ANOVA.

Next, we examined changes in LPAAT expression in murine primary skeletal muscle stem cells (satellite cells). When cultured in low serum differentiation media, satellite cells elongated and began to fuse within 1 day, and formed differentiated multinuclear myotubes within four days (**Fig. 2A**). We collected RNA extracts before (day 0) and at days 1 and 4 of differentiation and analyzed gene expression by RT-PCR. LPAAT3 mRNA was upregulated during differentiation and showed peak expression at day 1, while other LPAATs were unchanged or slightly decreased at day 1 and 4 time points (Fig. 2B). We also supplemented some of the culture media with 5 μM each of three fatty acids: LA (18:2n-6), AA (20:4n-6), and DHA (22:6n-3). We have used this fatty acid mixture previously to supply substrates for changes in cellular phospholipid compositions (11). Unexpectedly, inclusion of this fatty acid mixture in the culture media enhanced LPAAT3 expression during differentiation at the day 4 time point (Fig. 2B), but did not affect levels of other LPAATs.

Fatty acids are known ligands for PPARs (24, 25), and LPAAT3 was previously shown to be regulated by PPARα in cardiac muscle (21, 26). To examine whether the fatty acid mixture might enhance PPAR transcriptional activity in our culture system, we measured expression levels of two known PPAR-target genes, UCP3 and PDK4. These genes promote oxidative metabolism and have previously been shown to be PPARδ-regulated in skeletal muscle cells (27). Levels of both genes increased by day 4 of differentiation, and this was strongly enhanced by the fatty acid mixture (Fig. 2C). We also supplied the fatty acids individually during differentiation to determine which had activity to enhance LPAAT3 expression. Either DHA or AA applied at 5 μM enhanced LPAAT3 mRNA levels at the day 4 time point (Fig. 2D), which may reflect potencies of these fatty acids to stimulate PPARs (24) or other fatty acid-sensing cellular targets.

Effects of the fatty acid mixture (LA, AA, and DHA; 5 μM each) on LPAAT3 protein levels were also examined. LPAAT3 protein increased during satellite cell differentiation, and supplementation with the fatty acid mixture enhanced LPAAT3 protein levels at the day 4 time point (Fig. 2E). While LPAAT3 mRNA peaked at day 1 of differentiation even in the absence of fatty acids (Fig. 2B), fatty acid supplementation enhanced LPAAT3 mRNA and protein levels at day 4 rather than day 1 (Fig. 2B, E). This suggests that LPAAT3 may be subject to dual regulation; by the myogenic transcriptional program (at day 1) and at later time points by fatty acid-sensing pathways that might also regulate metabolic gene expression (Fig. 2C).

### Changes in cellular phospholipids during satellite cell differentiation

Although maintained at relatively low levels in cells, PA is a required precursor in the biosynthesis of all glycerophospholipids including PC and PE, the two most abundant phospholipids of cellular membranes. In order to detect changes in fatty acid compositions of cellular phospholipids that might result from altered LPAAT activities, satellite cells were cultured in growth or differentiation media supplemented with the fatty acid mixture (LA, AA, and DHA; 5 μM each), and cell extracts were collected after 2 days of culture for analyses by LC-MS. We performed comprehensive LC-SRM-MS analyses in positive ion mode with transitions to detect all PC and PE species containing two fatty chains 12–24 carbons in length. The peak areas of each species were normalized against the sum of all peak areas within that class to determine the relative abundances (expressed as percent of total PC or PE), and all major species comprising at least 2% of total PC or PE signals were plotted. Of note, plasmalogens, which are often rich in polyunsaturated fatty acids, were not detected at levels above the 2% threshold, possibly due to underestimation under our assay conditions (28).

**Fig. 3.**
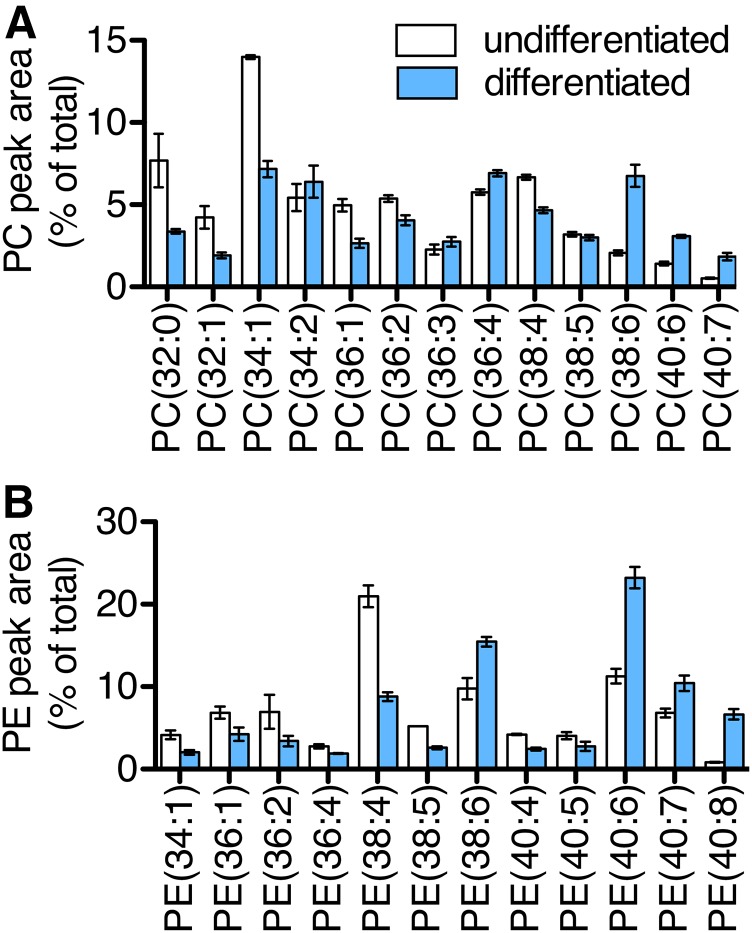
DHA-containing phospholipids increased during satellite cell differentiation. Satellite cells were incubated for 2 days in growth medium (undifferentiated) or differentiation medium (differentiated). Both media were supplemented with fatty acids (LA, AA, and DHA; 5 μM each). PC (A) and PE (B) species were measured by LC-MS. Peak areas are expressed as the percent of total PC or PE peak areas. Data represent the mean ± SEM from three independent experiments.

Relative levels of many PC and PE species were altered during differentiation (**Fig. 3A**, B). Several of the upregulated species, PC(38:6), PC(40:6), PC(40:7), PE(38:6), PE(40:6), PE(40:7), and PE(40:8), may contain DHA as one of their two fatty acid chains. LPAAT3 has been shown to selectively incorporate DHA into lysophosphatidic acid and promote production of DHA-containing phospholipids, and our group recently reported critical physiological roles for this activity in mouse testis and retina (11, 16, 29–31). In the present study, we observed that LPAAT3 levels increased concurrently with increases in several possible DHA-containing PC and PE species, suggesting that LPAAT3 may have functioned to promote DHA-containing phospholipid production during satellite cell differentiation.

### LPAAT3 knockdown decreased DHA incorporation during satellite cell differentiation

**Fig. 4.**
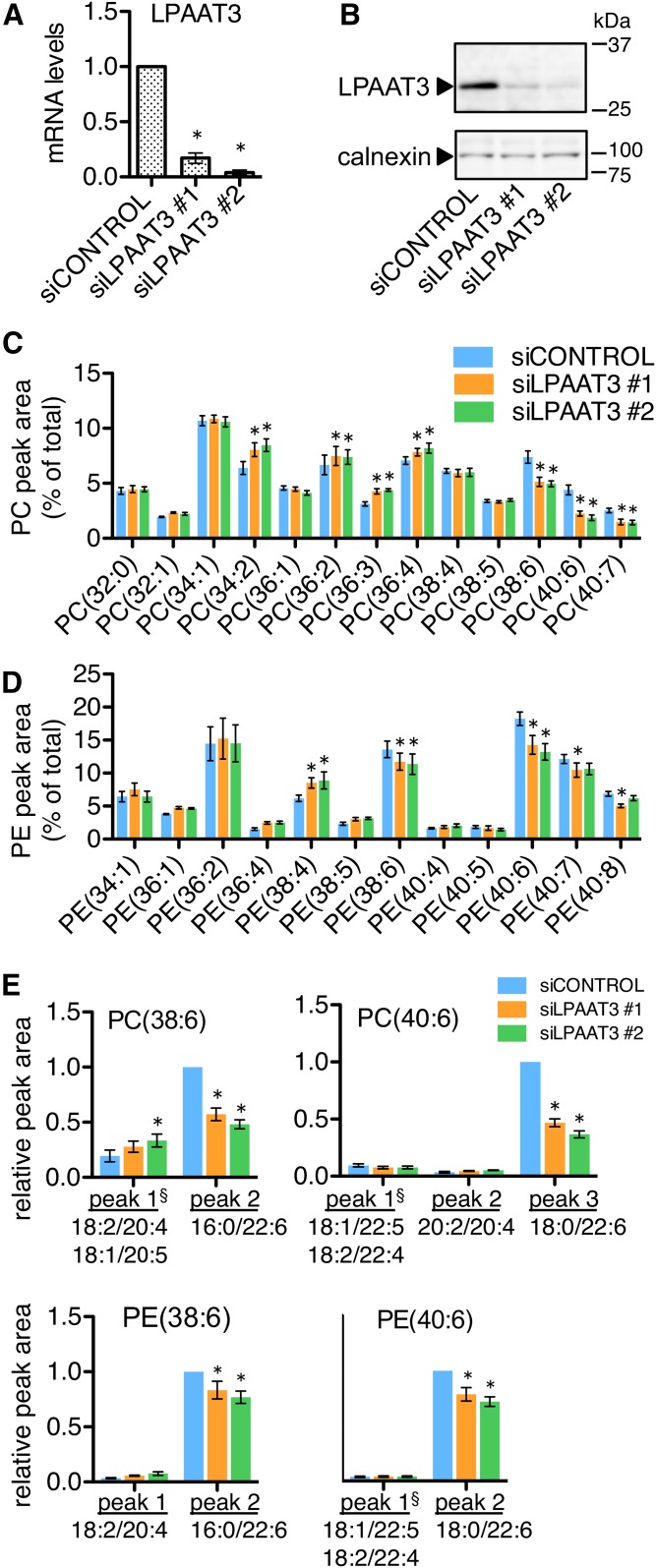
LPAAT3 knockdown reduced DHA-containing PC and PE during satellite cell differentiation. Satellite cells were transfected with siRNAs and then cultured for 2 days in differentiation medium containing the fatty acid supplement (LA, AA, and DHA; 5 μM each). A, B: siRNA efficiency. LPAAT3-targeting siRNAs (siLPAAT3 #1 and #2) reduced LPAAT3 mRNA (A) and protein levels (B) compared with nontargeting siRNA (siCONTROL). A representative Western blot from three independent experiments is shown; calnexin was detected as a loading control. C–E: LPAAT3 knockdown decreased DHA-containing phospholipids. PC (C) and PE (D) species were measured by LC-MS. Several possible DHA-containing species were decreased by LPAAT3 knockdown. E: DHA-containing isomers of PC(38:6), PC(40:6), PE(38:6), and PE(40:6) were selectively reduced by the LPAAT3-targeting siRNAs. For each of these species, chromatographic peak areas were normalized to total PC or PE signals and expressed relative to the highest peak in the control siRNA sample. Acyl chain set compositions of the chromatographic peaks were determined by fragmentation in negative ion mode (Table 2, supplemental Fig. S1). All data represent the mean ± SEM of three or four independent experiments. *P < 0.05, control-siRNA versus LPAAT3-siRNA; one-way ANOVA (A) or two-way ANOVA (C–E). ^§^Chromatographic peak containing mix of isomers.

We used gene-targeting siRNAs to directly assess the effects of LPAAT3 levels on the fatty acid compositions of cellular phospholipids during satellite cell differentiation. Either of two individual LPAAT3-targeting siRNAs (siLPAAT3 #1 or siLPAAT3 #2) or a nontargeting control siRNA (siCONTROL) was applied to cells at the start of differentiation. The fatty acid mixture (LA, AA, and DHA; 5 μM each) was also included in the differentiation medium, and cell extracts were collected after 2 days of differentiation. siLPAAT3 #1 and siLPAAT3 #2 were effective in reducing LPAAT3 mRNA and protein levels (**Fig. 4A**, B). Relative abundances of PC and PE species were analyzed by LC-MS. Several of the possible DHA-containing PC and PE species that had been upregulated during differentiation tended to be decreased by LPAAT3 knockdown (Fig. 4C, D). Additional targeted LC-MS/MS analyses were performed in order to determine the acyl chain set identities of PC(38:6), PC(40:6), PC(40:7), PE(38:6), PE(40:6), and PE(40:7). When detected in positive ion mode, each of these species resolved as two or three chromatographic peaks with differing retention times. In every case, negative ion mode fragment signals identified the predominant chromatographic peak as the DHA-containing isomer, while minor peaks contained other chain sets (**Table 2**, supplemental Fig. S1). For PC(38:6), PC(40:6), PE(38:6), and PE(40:6), LPAAT3 knockdown selectively reduced the DHA-containing chromatographic peaks (Fig. 4E). These results support that LPAAT3 functioned to incorporate DHA into phospholipids during satellite cell differentiation.

**TABLE 2.** Summary of identification of DHA-containing PC and PE species

Main Peak	Positive Ion Mode		Negative Ion Mode		Identification
	Precursor Ions (*m/z*)	Product Ions (*m/z*)	Precursor Ions (*m/z*)	Product Ions (*m/z*)	
PC(38:6) peak 2	806.6	184	866.6	255.1, 327.1	PC(16:0/22:6)
PC(40:6) peak 3	834.6	184	894.6	283.1, 327.1	PC(18:0/22:6)
PC(40:7) peak 3	832.6	184	892.6	281.1, 327.1	PC(18:1/22:6)
PE(38:6) peak 2	764.6	623.6	762.6	255.1, 327.1	PE(16:0/22:6)
PE(40:6) peak 2	792.6	651.6	790.6	283.1, 327.1	PE(18:0/22:6)
PE(40:7) peak 1	790.6	649.6	788.6	281.1, 327.1	PE(18:1/22:6)

Fatty acid compositions were determined in negative ion mode. The major chromatographic peak in each species was the DHA-containing isomer. Representative chromatograms and fatty acid identifications are shown in supplemental Fig. S1.

### PPARδ agonist GW1516 enhances LPAAT3 expression during satellite cell differentiation

**Fig. 5.**
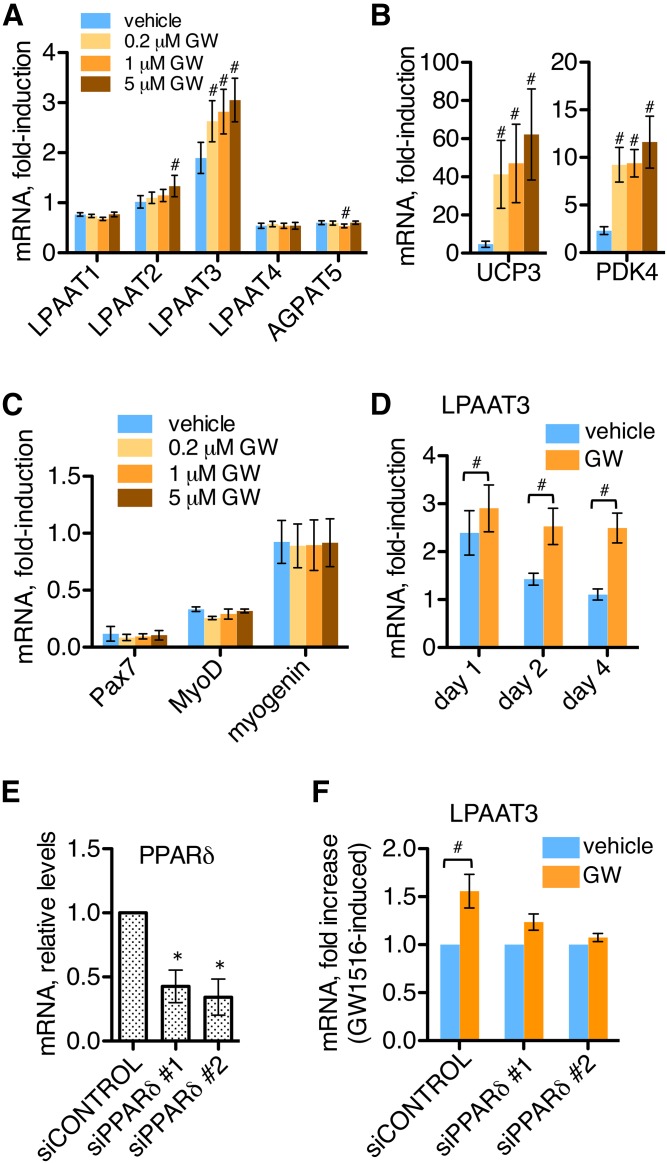
The PPARδ agonist GW1516 enhanced LPAAT3 expression during satellite cell differentiation. mRNA expressions were measured by RT-PCR; culture media were not supplemented with fatty acids. A–C: Effects of GW1516 (0.2, 1, or 5 μM) on gene expression at day 2 of differentiation. A: LPAAT3 expression was enhanced by GW1516 at each concentration; LPAAT2 was also slightly upregulated at the highest concentration. GW1516 upregulated PPAR-target genes UCP3 and PDK4 (B), but did not affect levels of myogenic marker genes Pax7, MyoD, and myogenin (C). D: GW1516-treatment time course. Satellite cells were incubated with vehicle or 1 μM GW1516 during 4 days of differentiation. GW1516 enhanced LPAAT3 expression more at later time points. E, F: GW1516-induced LPAAT3 expression is PPARδ dependent. PPARδ-targeting (siPPARδ #1 or siPPARδ #2) or nontargeting (siCONTROL) siRNAs were applied for 16 h, and then cells were differentiated for 2 days. E: siRNA efficiency. PPARδ-targeting siRNAs reduced PPARδ levels relative to nontargeting siRNA. F: PPARδ knockdown blocked GW1516-induced LPAAT3 expression; 1 μM GW1516 or vehicle was applied during 2 days differentiation. All data represent the mean ± SEM of three or four independent experiments; one-way ANOVA (A–C, E) or *t*-tests (D, F) were performed. ^#^*P* < 0.05, GW1516 versus vehicle; *P < 0.05, siCONTROL versus PPARδ siRNA.

LPAAT3 mRNA levels were enhanced by fatty acid supplementation during satellite cell differentiation, whereas expression levels of other LPAATs were not affected (Fig. 2B). Fatty acid supplementation also increased levels of UCP3 and PDK4 (Fig. 2C), which are known to be PPARδ-regulated in skeletal muscle (27, 32). To determine whether LPAAT3 might also be PPARδ-regulated in skeletal muscle, we treated satellite cell cultures with the PPARδ-selective agonist GW1516 at the start of differentiation, and effects on LPAAT expressions were assessed at day 2 of differentiation (**Fig. 5A**). LPAAT3 expression was enhanced by GW1516 treatment at all concentrations tested (0.2, 1, or 5 μM). LPAAT2 was also slightly increased in response to 5 μM GW1516, while levels of other LPAATs were not strongly affected. UCP3 and PDK4 were upregulated by all concentrations of GW1516 (Fig. 5B), while levels of the myogenic marker genes Pax7, MyoD, and myogenin were not affected (Fig. 5C).

Satellite cells were also treated with vehicle or GW1516 (1 μM) over a time course of 4 days of differentiation. In the presence of vehicle, LPAAT3 mRNA showed peak expression at day 1 of differentiation; and LPAAT3 expression was enhanced by GW1516, with greater enhancements observed at the later time points (Fig. 5D). To confirm the specificity of the PPARδ agonist, we transfected cells with two different PPARδ-targeting siRNAs to suppress PPARδ cellular levels (Fig. 5E). PPARδ knockdown by either siRNA also suppressed the GW1516-induced increase in LPAAT3 expression (Fig. 5F). These results confirm the specificity of GW1516 for PPARδ and indicate that LPAAT3 was PPARδ-regulated in differentiating satellite cells. Additionally, LPAAT3 expression at day 1 was not as strongly affected by GW1516 as at days 2 and 4 (Fig. 5D), suggesting that early LPAAT3 induction may be PPARδ independent and occur as part of the myogenic differentiation program.

### GW1516 and AICAR enhance LPAAT3 expression and DHA incorporation into phospholipids during satellite cell differentiation

The AMPK agonist AICAR has been shown to stimulate oxidative metabolic gene expression in cultured skeletal muscle cells and, when applied together with GW1516, synergistically promoted enhanced expressions of UCP3 and PDK4 (27). We applied GW1516 and AICAR individually or in combination to differentiating satellite cells and examined the effects on the expression of LPAAT3, UCP3, and PDK4. The cells were allowed to differentiate for 2 days prior to treatment, and then differentiated for an additional 2 days in the presence of drug treatments. Media were supplemented with the fatty acid mixture during the final 2 days of differentiation.

**Fig. 6.**
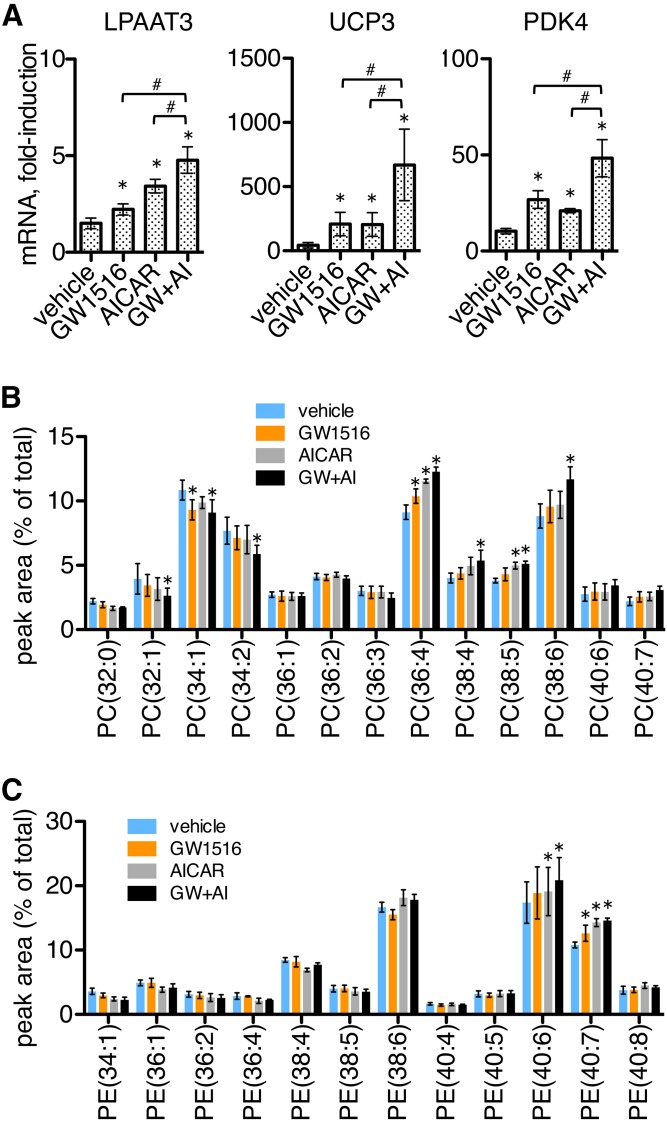
GW1516 and AICAR stimulated LPAAT3, UCP3, and PDK4 expression and promoted increased phospholipid-DHA content in differentiated satellite cells. Cells were differentiated for 2 days and then differentiated for an additional 2 days in the presence of vehicle, GW1516 (1 μM), and/or AICAR (1 mM). Media were supplemented with fatty acids (LA, AA, and DHA; 5 μM each) during the final 2 days of differentiation. A: Expressions of LPAAT3, UCP3, and PDK4 were enhanced by GW1516, AICAR, and combination (GW+AI) treatments. Gene levels were measured by RT-PCR and are expressed as fold-induction following differentiation. Data represent the mean ± SEM of three independent experiments. *P < 0.05, drug versus vehicle; ^#^*P* < 0.05, single versus both drugs; one-way ANOVA. B, C: Treatment with GW1516 and AICAR increased several DHA-containing phospholipids. PC (B) and PE (C) species were measured by LC-MS. Data represent the mean ± SEM of three independent experiments. *P < 0.05, drug versus vehicle; two-way ANOVA.

Treatment with either AICAR or GW1516 increased expression of LPAAT3, and expression was further enhanced when the compounds were applied together. Similar patterns of increases were also observed for the expressions of UCP3 and PDK4 (**Fig. 6A**). We performed LC-MS analyses to measure relative abundances of PC and PE species under the same conditions (Fig. 6B, C). Several PC species were affected, and either GW1516 or AICAR tended to increase levels of several polyunsaturated fatty acid-containing PC species: PC(36:4), PC(38:4), and PC(38:5). This was more pronounced by combination treatment, which also increased the DHA-containing species PC(38:6). DHA-containing PE species were also upregulated by drug treatments. GW1516 treatment increased levels of PE(40:7), and either AICAR or combination treatments increased levels of both PE(40:6) and PE(40:7) (Fig. 6C). These results show that treatment with GW1516 and AICAR enhanced LPAAT3, PDK4, and UCP3 expression in differentiating satellite cell cultures, and cotreatment with both drugs promoted increased incorporation of DHA into several PC and PE species. AICAR administration has been shown to promote endurance in mice, which is potentiated by pharmacological PPARδ activators and accompanied by transcriptional changes in skeletal muscle, including upregulation of PDK4 and UCP3 (27, 33). Our results indicate that LPAAT3 was upregulated by the same exercise-induced pathways during in vitro differentiation, suggesting that LPAAT3 might also contribute to enhanced phospholipid-DHA content in endurance-trained muscle.

## DISCUSSION

Skeletal muscle can adapt its metabolism to meet physiological demands, and increased DHA content in phospholipids is reported to occur as part of the adaptive response to endurance exercise (4–7). Our group and others have identified several LPAATs that incorporate fatty acids into cellular phospholipids based on their substrate selectivities and generate diversity in cellular membranes (34). In the present study, we identified LPAAT3 as an enzyme that was upregulated during myoblast differentiation and functioned to promote incorporation of DHA into PC and PE. LPAAT3 expression was enhanced by fatty acids, the PPARδ agonist GW1516, and the AMPK activator AICAR. AICAR and GW1516 are exercise-mimicking drugs that promote an endurance phenotype (1), suggesting that LPAAT3 may also have a role to enhance phospholipid DHA-content of skeletal muscle as a part of the adaptive response to endurance training.

The molecular mechanisms that mediate enhanced phospholipid DHA levels in endurance-trained muscle are not well known. The nuclear receptor coactivator PGC1α is activated by exercise to mediate mitochondrial biogenesis and enhanced oxidative capacity in skeletal muscle. Senoo et al. (4) reported that PGC1α overexpression in skeletal muscle resulted in altered phospholipid profiles, with the biggest alterations being increased PC(18:0/22:6) and PE(18:0/22:6) in glycolytic EDL muscles, and these same alterations also occurred after exercise training in a PGC1α-dependent manner. In the current study, we found that the corresponding species, PC(40:6) and PE(40:6), were increased during satellite cell differentiation concurrently with enhanced LPAAT3 levels (Figs. 2B; 3A, B). LC-MS/MS fragment ion analyses revealed that both of these species were comprised primarily of DHA-containing isomers, and these were specifically decreased by LPAAT3 siRNAs (Fig. 4C–E). PGC1α is activated downstream of AMPK and is a coactivator of PPARδ (32). Our finding that LPAAT3 and DHA incorporation were upregulated by GW1516 and AICAR (Fig. 6A–C) suggests that enhanced LPAAT3 expression might be a common mechanism contributing to increased DHA incorporation into PC and PE via convergent PPARδ and AMPK/PGC1α pathways.

DHA is widely taken as a dietary supplement and may have broad beneficial metabolic effects; however, the effects of exercise-induced enhanced phospholipid-DHA content on skeletal muscle metabolism are not well understood. Oxidative capacity and oxidative fiber content positively correlate with phospholipid-DHA levels, suggesting a positive effect on oxidative capacity. Senoo et al. (4) observed that exercise training increased phospholipid-DHA in glycolytic EDL muscle of mice, whereas untrained soleus muscle (oxidative) already had high phospholipid-DHA content. In other studies, PE-DHA content was reported to be much higher in oxidative than in glycolytic vastus lateralis muscles of rats (8), and enhanced phospholipid-DHA content correlated with enhanced type I oxidative fiber percentage in vastus lateralis muscles of human endurance athletes (7). Together these studies support a consensus that phospholipid-DHA content positively correlates with oxidative capacity and may be increased during conversion of glycolytic to more oxidative fiber types.

Enhanced DHA in skeletal muscle might impact cellular metabolism by several mechanisms. Due to its high number of double bonds, increased DHA content is predicted to impart increased curvature and fluidity to membranes, and this may affect functions of cellular organelles (35). However, few studies have addressed the functional impact of enhanced DHA content in mitochondrial membranes on mitochondrial energetics. In one study, 12 weeks of fish oil supplementation in active men resulted in pronounced incorporation of DHA and EPA into PC and PE of skeletal muscle mitochondrial membranes. No changes in maximal mitochondrial respiration were detected, but improvements in several parameters of respiratory function occurred, including increases in mitochondrial ADP sensitivity, submaximal ADP-stimulated respiration, and maximal mitochondrial ROS emission (36). However, whether these changes were due to altered biophysical properties of mitochondrial membranes or other mechanisms is unclear because free fatty acid DHA or EPA may also strongly impact metabolic gene expression through activation of lipid-sensing transcription factors such as PPARs and other molecules (37–39). Because skeletal muscle DHA levels may affect metabolism either through changes in metabolic gene expression or altered biophysical properties of cellular membranes, it is important to identify the enzymes and mechanisms that govern partitioning of DHA between phospholipid and free fatty acid form.

**Fig. 7.**
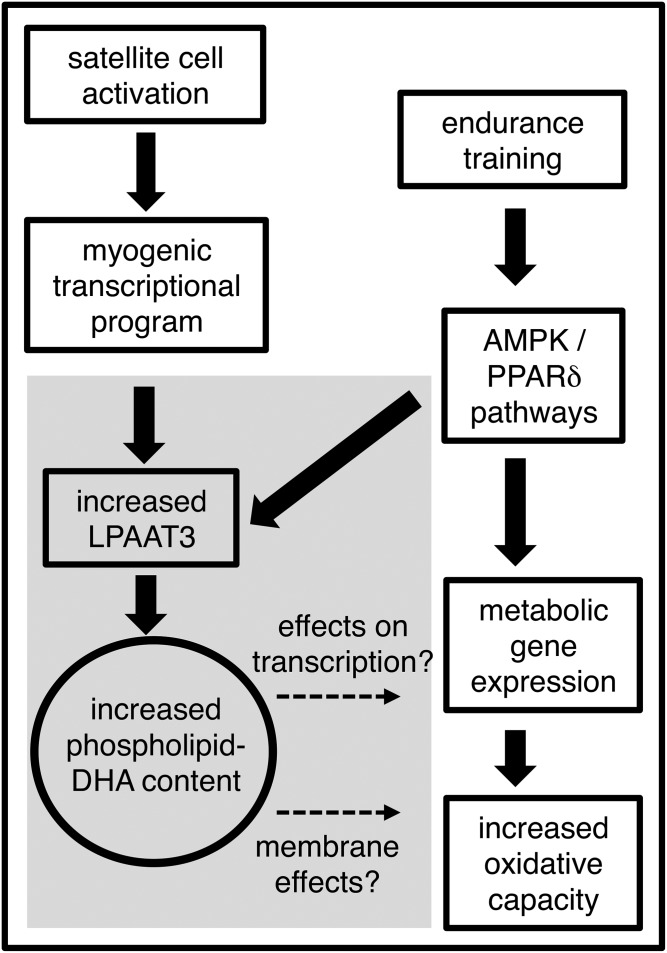
Proposed mechanism and function of enhanced DHA content in endurance-trained skeletal muscle. LPAAT3 levels may increase in activated satellite cells as part of the myogenic transcriptional program. Exercise-activated AMPK and PPARδ pathways, which stimulate metabolic gene expression, may also enhance LPAAT3 levels in myofibers. DHA incorporation into membranes may alter their biophysical properties and thereby affect cellular metabolism. DHA incorporation into membranes may also impact free DHA levels to influence activities of lipid-sensing transcription factors such as PPARs. Findings from this study are in the shaded region; dashed arrows indicate possible metabolic effects of the enhanced phospholipid-DHA content.

Here, we identify LPAAT3 as an enzyme that incorporated DHA into phospholipids of skeletal muscle cells and that might also contribute to the enhanced DHA levels in endurance-trained muscles. Recently LPAAT3 has been reported to have an essential role in the incorporation of DHA into phospholipids of several tissues, including retina and testis (30, 31). LPAAT4 has also been reported to be involved in production of DHA-containing phospholipids (19), and additional enzymes may also incorporate DHA at lower levels. It is likely that regulation of DHA levels in cellular membranes of various tissues is complex and determined by combinations of factors that include phospholipid turnover, competing activities of several enzymes, and substrate availabilities. We propose that LPAAT3 upregulated in activated satellite cells or during adaptation of skeletal muscle to endurance training may have a unique role to increase phospholipid-DHA levels, with possible effects on metabolism related to effects on gene transcription or membrane properties (**Fig. 7**). Further identification of the enzymes and mechanisms that regulate DHA content in skeletal muscle will enhance our understanding of the broad metabolic effects of DHA and related health benefits associated with dietary omega-3 fatty acids and exercise.

## Supplementary Material

Supplemental Data
