# Supplementary material for: LPAAT3 incorporates docosahexaenoic acid into skeletal muscle cell membranes and is upregulated by PPARδ activation
Source: J Lipid Res. 2017 Dec 28;59(2):184–94. doi: 10.1194/jlr.M077321 (PMC5794415; doi:10.1194/jlr.M077321)
Supplement: Supplemental Data [file supp_59_2_184__index.html]

Lysophosphatidic Acid Acyltransferase 3 Incorporates Docosahexaeneoic Acid into Skeletal Muscle Cell Membranes and Is Upregulated by PPARδ Activation — LPAAT3 incorporates docosahexaenoic acid into skeletal muscle cell membranes and is upregulated by PPARδ activation — Supplemental Data 

# LPAAT3 incorporates docosahexaenoic acid into skeletal muscle cell membranes and is upregulated by PPARδ activation

## Supplemental Data

- Supplemental figure S1 (.pdf, 266 KB) - Fatty acid chain compositions of possible DHA-containing PC and PE species.
