# Supplementary material for: LPAAT3 incorporates docosahexaenoic acid into skeletal muscle cell membranes and is upregulated by PPARδ activation
Source: J Lipid Res. 2017 Dec 28;59(2):184–94. doi: 10.1194/jlr.M077321 (PMC5794415; doi:10.1194/jlr.M077321)
Supplement: Supplemental Data [file 10.1194_M077321_jlr.M077321-1.pdf]

SUPPLEMENTAL INFORMATION

Lysophosphatidic Acid Acyltransferase 3 Incorporates Docosahexaeneoic Acid into Skeletal Muscle Cell Membranes and Is Upregulated by PPARδ Activation

William J. Valentine[*], Suzumi M. Tokuoka[¶], Daisuke Hishikawa[*], Yoshihiro Kita[¶†], Hideo Shindou[*#§1], and Takao Shimizu[*¶]

[*]Department of Lipid Signaling, National Center for Global Health and Medicine, Shinjuku-ku, Tokyo 162-8655, Japan

[¶]Department of Lipidomics, [†] Life Sciences Core Facility, and [§]Department of Lipid Science, Graduate School of Medicine, The University of Tokyo, Bunkyo-ku, Tokyo 113-0033, Japan

[#]AMED, Chiyoda-ku, Tokyo 100-0004, Japan

supplementary figure S1

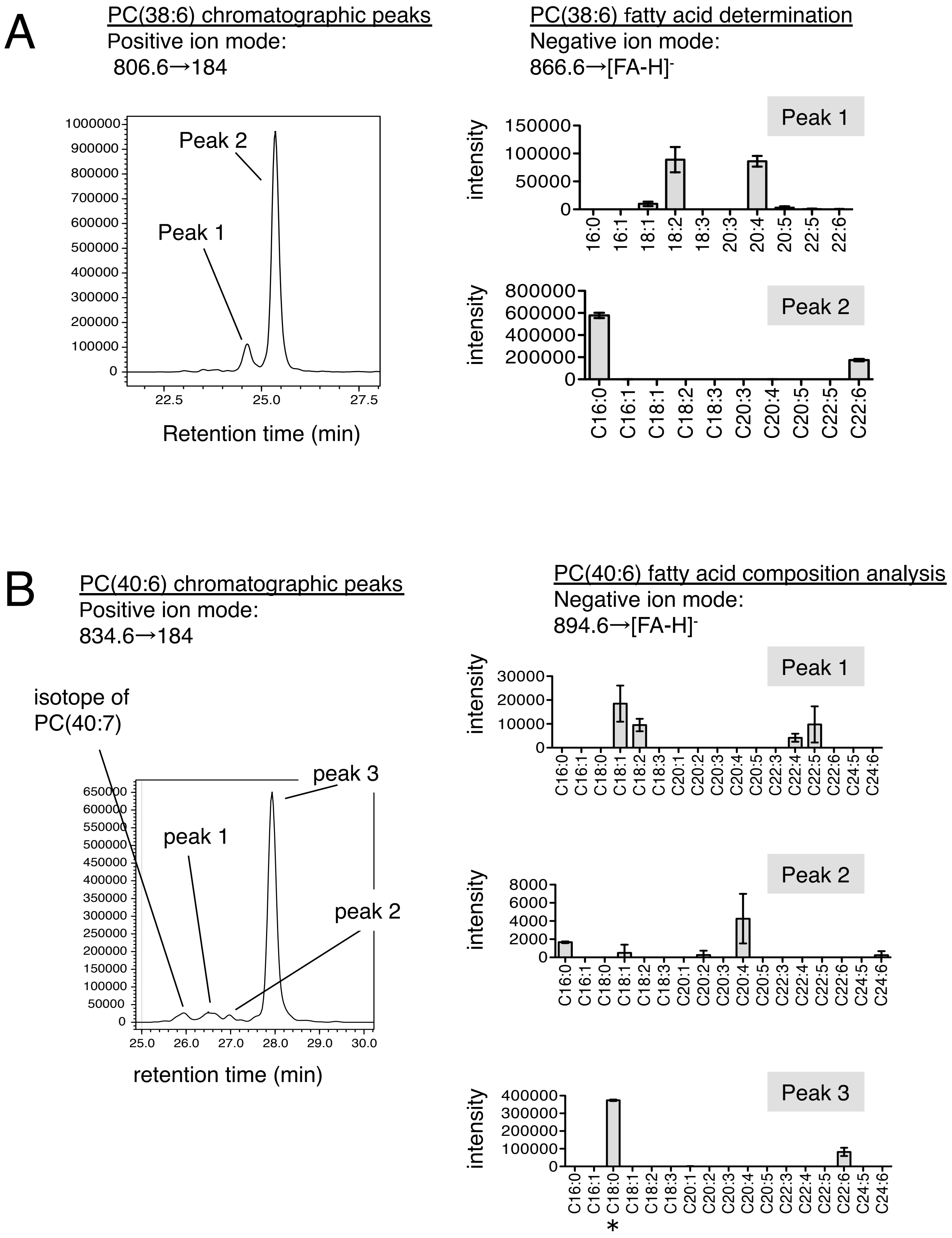

supplementary figure S1

C

PC(40:7) chromatographic peaks
Positive ion mode:
832.6→184

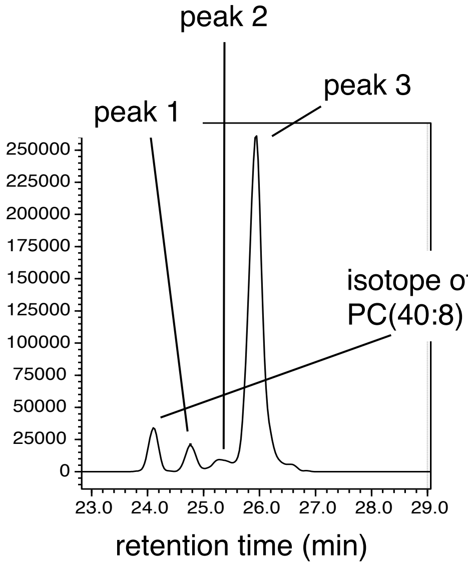

PC(40:7) fatty acid determination
Negative ion mode:
892.6→[FA-H]$^-$

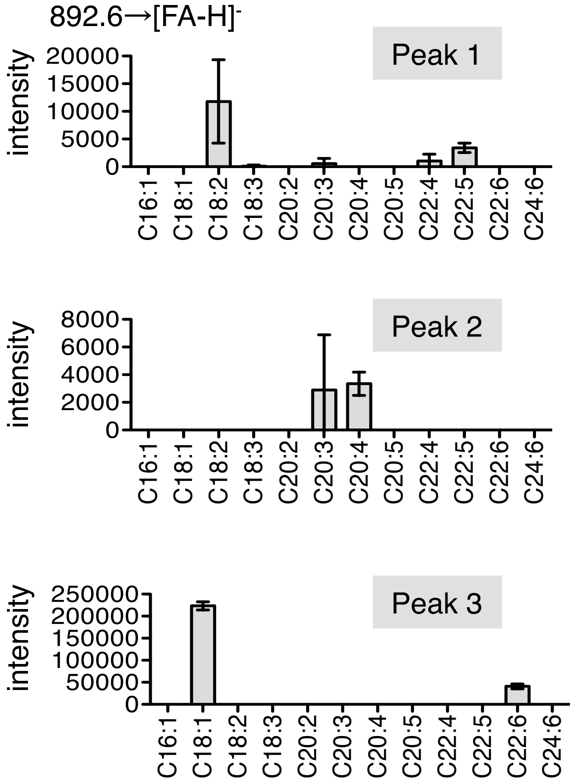

D

PE(38:6) chromatographic peaks
Positive ion mode:
764.6→623.6

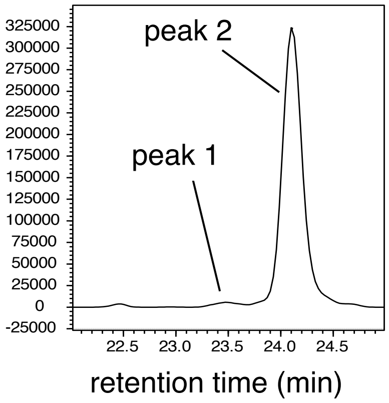

PE(38:6) fatty acid determination
Negative ion mode:
762.6→[FA-H]$^-$

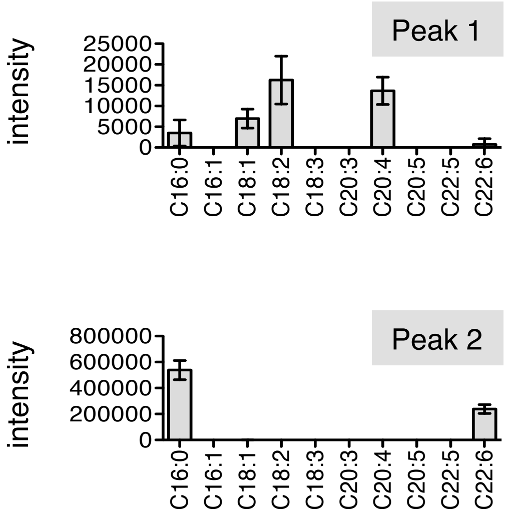

supplementary figure S1

E

PE(40:6) chromatographic peaks
Positive ion mode:
792.6→651.6

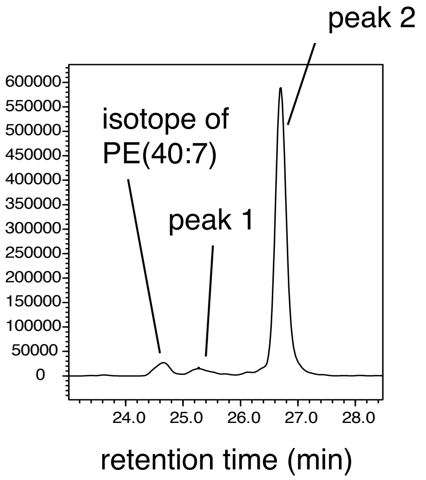

PE(40:6) fatty acid determination
Negative ion mode:
790.6→[FA-H]$^-$

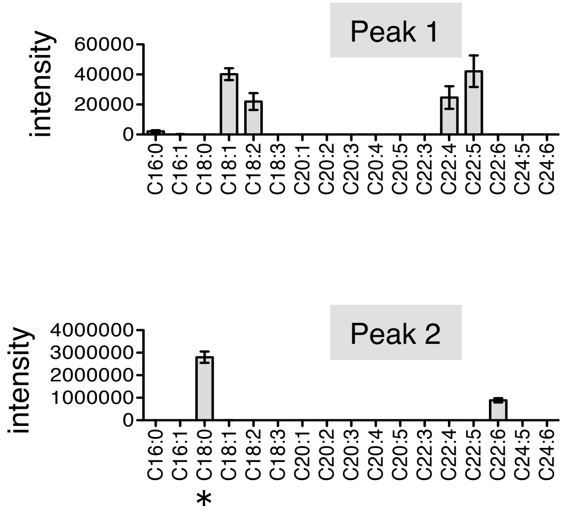

F

PE(40:7) chromatographic peaks
Positive ion mode:
790.6→649.6

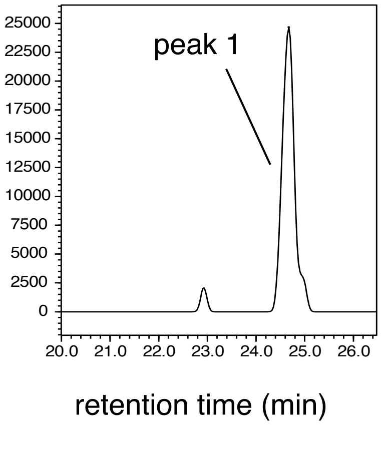

PE(40:7) fatty acid determination
Negative ion mode:
788.6→[FA-H]$^-$

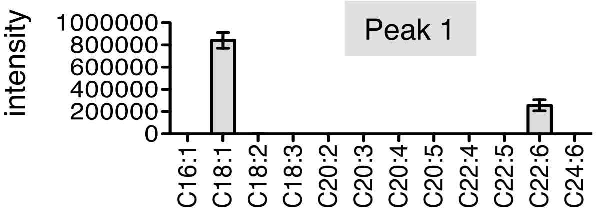

Supplemental figure S1. Fatty acid chain compositions of possible DHA-containing PC and PE species. Satellite cells were transfected with control siRNAs and differentiated for 2 days in media supplemented with fatty acids (LA, AA, and DHA; 5 μM/each). A–F: Various PC and PE species – PC(38:6) (A), PC(40:6) (B), PC(40:7) (C), PE(38:6) (D), PE(40:6) (E), and PE(40:7) (F) – were measured by LC-MS in both positive ion mode and negative ion mode. The chromatographic peaks in positive ion mode with differing retention times were obtained with the following transitions: $[M+H]^+ \rightarrow 184$ for PC (A–C, left panels) and $[M+H]^+ \rightarrow [M+H\text{-}141]^+$ for PE (D–F, left panels). Negative ion mode fragment ion signals of possible fatty acyl chains were obtained using the following transitions: $[M+HCO3]^- \rightarrow [FA\text{-}H]^-$ for PC (A–C, right panels) and $[M\text{-}H]^- \rightarrow [FA\text{-}H]^-$ for PE (D–F, right panels), where [FA] is the monoisotopic mass of the fatty acid. The following acyl chains were targeted: C16:0 (m/z 255.1), C16:1 (m/z 253.1), C18:0 (*m/z 283.1), C18:1 (m/z 281.1), C18:2 (m/z 279.1), C18:3 (m/z 277.1), C20:1 (m/z 309.1), C20:2 (m/z 307.1), C20:3 (m/z 305.1), C20:4 (m/z 303.1), C20:5 (m/z 301.1), C22:3 (m/z 333.1), C22:4 (m/z 331.1), C22:5 (m/z 329.1), C22:6 (m/z 327.1), C24:5 (m/z 357.1) and C24:6 (m/z 355.1). Error bars are SD (n=3). *: May also contain signals of decarboxylated C22:6 (DHA) in addition to signals of C18:0.
